# Selection of patient-reported outcome measures (PROMs) for use in health systems

**DOI:** 10.1186/s41687-021-00374-2

**Published:** 2021-10-12

**Authors:** Fatima Al Sayah, Xuejing Jin, Jeffrey A. Johnson

**Affiliations:** 1 Alberta PROMs & EQ-5D Research & Support Unit (APERSU), School of Public Health, University of Alberta, 2-040 Li Ka Shing Centre for Health Research Innovation, Edmonton, AB T6G 2E1 Canada; 2 Centre for Evidence-Based Medicine, School of Traditional Chinese Medicine, Beijing University of Chinese Medicine, Beijing, China

## Abstract

Many healthcare systems around the world have been increasingly using patient-reported outcome measures (PROMs) in routine outcome measurement to enhance patient-centered care and incorporate the patient’s perspective in health system performance evaluation. One of the key steps in using PROMs in health systems is selecting the appropriate measure(s) to serve the purpose and context of measurement. However, the availability of many PROMs makes this choice rather challenging. Our aim was to provide an integrated approach for PROM(s) selection for use by end-users in health systems.

The proposed approach was based on relevant literature and existing guidebooks that addressed PROMs selection in various areas and for various purposes, as well as on our experience working with many health system users of PROMs in Canada. The proposed approach includes the following steps: (1) Establish PROMs selection committee; (2) Identify the focus, scope, and type of PROM measurement; (3) Identify potential PROM(s); (4) Review practical considerations for each of the identified PROMs; (5) Review measurement properties of shortlisted PROMs; (6) Review patient acceptance of shortlisted PROMs; (7) Recommend a PROM(s); and (8) Pilot the selected PROM(s). The selection of appropriate PROMs is one step in the successful implementation of PROMs within health systems, albeit, an essential step. We provide guidance for the selection of PROMs to satisfy all potential usages at the micro (patient-clinician), meso (organization), and macro (system) levels within the health system. Selecting PROMs that satisfy all these purposes is essential to ensure continuity and standardization of measurement over time. This is an iterative process and users should consider all the available information from all presented steps in selecting PROMs. Each of these considerations has a different weight in diverse clinical contexts and settings with various types of patients and resources.

## Background

Many healthcare systems around the world have been increasingly using patient-reported outcome measures (PROMs) in routine outcome measurement to enhance patient-centered care and incorporate the patient’s perspective in health system performance evaluation [1–6]. Hundreds of PROMs are available and there are many more in development [7]. One of the key steps in using PROMs in health systems is selecting the appropriate measure(s) to serve the purpose and context of measurement. However, the availability of many PROMs makes this choice rather challenging.

Unlike their application in comparative effectiveness research or population health surveillance, the use of PROMs in health systems is intended to serve multiple purposes for several users [2, 3]. At the micro level, PROMs are used by clinicians to support individual patients’ clinical management. At the meso level, PROMs data are aggregated and used by administrators and managers to evaluate programs and inform service delivery and quality improvement initiatives at an organization level. At the macro level, aggregated PROMs data are used by executives and policy-makers for health system performance evaluation and for public reporting. This poses a challenge on the process of selecting a PROM that could satisfy all these potential purposes and users’ needs.

An important consideration in the use of PROMs in health systems is the standardization and consistency of measurement across the system [8]. For example, in England, the National Health Service (NHS) initiated the first system-level use of PROMs in four elective surgeries under the NHS PROMs program [5]. The EQ-5D is used as a generic PROM across all surgeries, alongside a condition-specific PROM for each clinical area. In Sweden, national quality registers use EQ-5D as a generic PROM across all registers, alongside condition-specific PROMs in some registers [6]. In Canada, the Edmonton Symptom Assessment Scale, a generic PROM of symptom burden, is used across all cancer care centers [9], and a modified version for renal care is collected in many hemodialysis units [10, 11]. In the province of Alberta, the EQ-5D-5L is used as a generic PROM in routine outcome measurement across the healthcare system [2]. This standardization in measurement is crucial as it allows generating population norms and setting benchmarks against which to compare PROMs in specific clinical groups.

The need to serve multiple purposes and users and the importance of consistency and standardization in measurement make the selection of PROMs for use within health systems a crucial component of successful PROMs programs. Previous literature focused on providing guidance for selecting PROM(s) for research purposes in specific clinical areas [12–15]. In this paper, we expand on previous work and provide an integrated approach for PROM(s) selection for use in health systems.

## Taxonomy of PROMs

PROMs could be categorized in several ways; here we provide a taxonomy of PROMs based on the focus, scope, and type of measurement.

### Focus of measurement

Health outcomes are broadly defined as changes in health status that occur as a result of a health-related event or illness, or a healthcare intervention. Some health outcomes are patient-reported and are measured using PROMs; these include outcomes such as symptom burden (e.g. pain, dizziness), functional status (e.g. physical functioning) and health-related quality of life [16]. Identifying a patient-reported outcome of interest is crucial to PROMs selection. In health system applications, it is important to consider overall health as an outcome of interest given its usefulness in performance evaluations and comparative analyses [17]. Nonetheless, other outcomes, such as symptom burden and disease-specific quality of life, could be considered.

### Scope of measurement

PROMs could be categorized as generic or condition-specific. Generic PROMs are designed to assess general aspects of health that are not specific to a particular disease (e.g., EQ-5D, SF-36, PROMIS), while condition-specific PROMs assess aspects of health specific to a disease (e.g., WOMAC, EORTC-QLQ-C30) [18]. Generic and condition-specific PROMs each have their advantages and disadvantages. Generic PROMs are useful when comparing patients across different health conditions; however, changes in specific aspects of health are better assessed using condition-specific PROMs. Because of these advantages and disadvantages, it is often recommended to use a generic and a condition-specific PROM. In using a combination of generic and disease-specific measures, it is important for users to check and compare the domains or items in each of the measures, and avoid redundancy between the two measures which imposes undue respondent burden and potentially annoyance and reduce compliance with repeated assessment over time.

### Type of measurement

PROMs could be also categorized as profile or preference-based. Profile measures (e.g., SF-36, WHOQOL, PROMIS) are used to determine the position of some characteristic on specific domains, which are measured by multiple items and reported by domain [19]. Preference-based measures (e.g., EQ-5D, HUI) are used to place a value on health states or conditions, and typically yield a single multi-domain index score. Profile measures are useful in applications that aim to assess health or an aspect of it from a patient perspective in the evaluation of treatments or services. While preference-based measures could be used for these purposes as well, they are also used to generate utility values to calculate quality-adjusted life years for use in health economic evaluations.

## Selecting PROMs for use in health systems

The selection of PROMs requires a balance among several factors and pragmatic considerations. A health system may wish to establish a common measure, while also enabling a degree of flexibility for more tailored/specific PROM collection. The following approach is based on previous literature and guidebooks on PROMs selection [12–15, 17, 20–24] identified via a brief scoping review and included key and high quality articles on this topic, as well as our experience working with many health system users of PROMs in Canada, which helped identify important practical issues around PROMs selection. This approach to PROMs selection for use within health systems could be modified to align with contextual factors in certain settings or clinical areas. The following steps are presented in a sequential fashion; however, this process is iterative and users may need to go back and forth between the different steps until completion.

### Step 1: Establish PROMs selection committee

PROMs program managers should establish a PROMs selection committee that includes system administrators, PROMs methodologic experts, patient or care provider representative, clinicians, and policy-makers. The committee would be involved in all steps of the PROMs selection process, and would periodically review, deliberate and provide feedback on various aspects of the process.

### Step 2: Identify the focus, scope, and type of PROM measurement

The committee would identify the target population, which could be based on a clinical condition or procedure, or may include all patients within the system. Additionally, the committee should identify the focus of measurement, which may include one or more patient-reported outcomes (e.g., symptom burden, health-related quality of life), as well as the scope (generic, condition-specific), and type of measurements (profile, preference-based) to guide PROMs selection.

The type of PROM(s) to use depends on the purpose of measurement. In selecting PROM(s) for use in health systems, the measures need to satisfy two key purposes: use at the individual patient (i.e., micro-) level to support patient clinical management, and use at the aggregate (i.e., meso-, macro-) level to support performance evaluation, healthcare delivery planning, comparative effectiveness analysis, as well as economic and policy analyses. Given the multiple purposes and users of PROMs within the system, multiple measures may need to be identified to serve all these purposes.

From a patient perspective, PROMs represent an opportunity to report specific aspects of health or problems, which vary depending on the clinical context. While cancer patients may consider symptom burden an important outcome, physical functioning would be important for patients after a hip arthroplasty. Clinicians on the other hand focus on outcomes presented in clinical guidelines and tend to value condition-specific measures and symptom assessment scales given their clinical usefulness. Quality managers, administrators, and policy-makers value generic measures of overall health that allow comparisons across clinical conditions and performance evaluation at the system level, as well as the evaluation of health outcomes as they relate to health policy.

### Step 3: Identify potential PROM(s)

After identifying the focus and type of measurements, a scoping review of the literature to identify existing measures that fit the purposes of measurement, with a focus on their conceptual and measurement model, should be conducted. This review should focus on studies that developed, modified or validated a certain measure. Some generic PROMs such as the EQ-5D and SF-36, and condition or symptom-specific measures such as the EORTC-QLQ-C30 and PHQ-9 have a long legacy of use and are often identified by PROMs experts. A list of all identified measures is compiled and evaluated in the next step.

### Step 4: Review practical considerations

Practical considerations should be reviewed to shortlist the identified measures as these are determining factors for selecting PROMs in many contexts (Table 1). A key consideration is the ease of PROM integration into clinical workflow, which depends on the length of the measure (lengthy PROMs increase respondent burden and may lead to incomplete data), ease of administration (e.g., simple presentation of questions), and availability of various administration modes (e.g., paper-based, web, proxy) and languages. The choice of a collection mode depends on the proposed use of the data and availability of technology and resources for establishing collection and reporting systems. Consideration must also be given to regulatory expectations, data security and ownership, and ease of access and data interoperability, all of which are impacted by planned uses of the data.

**Table 1:** Practical considerations of PROMS implementation

Integration into clinical workflow	Length of measure
	Administration mode
	Timing and frequency of administration
Data and analysis	Technology and resources available
	Availability of country-specific value set
	Data security/access and ownership
Licensing fees	Budget considerations over time

The timing and frequency of PROM(s) administration, which also impacts the integration into clinical workflow, is also important. Timing and frequency of measurements depend on the clinical context. For example, when a specific intervention is delivered, such as surgeries, PROMs are often administered once before surgery and once or more afterwards, while patients with chronic illness may complete PROMs at each clinic visit. Users should also consider the reference period of the measure(s), which should align with the timing and frequency of measurements.

If a preference-based PROM is identified, users need to identify whether a country-specific value set is available. Finally, users need to consider licensing fees. While some PROMs are available in the public domain with published norms and scoring protocols, others are proprietary and fees associated with large-scale and long-term use within health systems need to be considered.

### Step 5: Review measurement properties

Once the identified measures are shortlisted, a review of their measurement properties is done. Although many validated PROMs have been in use for decades, they must be evaluated for appropriateness with specific patient populations and conditions. For use in the health system, there needs to be sufficient evidence on PROM(s) reliability, validity, responsiveness and interpretability as they are intended to assess an outcome at one time point and changes in the outcome over time. Guidance is available to inform such considerations, such as the “Terwee criteria” [25] and COSMIN guideline [21]. Users could also identify systematic reviews examining measurement properties of shortlisted PROMs.

### Step 6: Review patient acceptance

Given that patient representatives are members of the committee and are engaged in the selection process, patient acceptance would presumably be addressed in previous steps. However, it is important to consider some patient attributes that may influence PROMs selection such as language, literacy level, and cultural appropriateness, as well as other factors such as visual impairment, dexterity issues or other functional limitations that may affect patients’ ability to complete PROM(s). Patients or patient representatives who were not involved in the selection process could be consulted at this stage.

### Step 7: Recommend a PROM(s)

After careful assessment of practical considerations, measurement properties, and patient acceptance, the PROMs selection committee would recommend a PROM or multiple PROMs for use in the target population. For example, the use of a common, generic measure across the system, while allowing the flexibility of integrating another PROM(s) for a specific population, geographic area or other sub-population of interest.

### Step 8: Pilot the PROM(s)

Before large-scale use of the selected PROM(s) within the system, we encourage users to consider piloting the selected PROM(s) in the target setting. Evaluation measures, such as brief surveys, interviews and narratives among patients, users (collectors) and management should be included. This pilot would further inform patient and clinician acceptance of PROM(s), expose any challenges with integration into clinical workflow, and uncover other contextual issues that may have been missed during the selection process. Once complete, reports to the PROMS selection committee regarding the integration into clinical workflow, data analysis and value would strengthen the PROMs program and subsequent implementations.

The selection of appropriate PROMs is one step in the successful implementation of PROMs within health systems, albeit, an essential step. We provide guidance for the selection of PROMs to satisfy all potential usages at the micro (patient-clinician), meso (organization), and macro (system) levels within the health system. Selecting PROMs that satisfy all these purposes is essential to ensure continuity and standardization of measurement over time. This is an iterative process and users should consider all the available information from all presented steps in selecting PROMs. Each of these considerations has a different weight in diverse clinical contexts and settings with various types of patients and resources.

## Data Availability

Not applicable.

## Abbreviations

COSMIN: Consensus-based standards for the selection of health measurement instruments
EORTC-QLQ-C30: European Organization for the Research and Treatment of Cancer, quality of life questionnaire
EQ-5D-5L: EuroQol, 5-dimension, 5-level questionnaire
HUI: Health utility index
NHS: National Health Service
PHQ-9: Patient health questionnaire, 9-items
PROMIS: Patient-reported outcomes measurement information system
PROMs: Patient-reported outcome measures
SF-36: 36-Item, Short-form health survey
WHOQOL: World Health Organization quality of life assessment
WOMAC: Western Ontario and McMaster universities arthritis index
